# Pooled Sequencing and Rare Variant Association Tests for Identifying the Determinants of Emerging Drug Resistance in Malaria Parasites

**DOI:** 10.1093/molbev/msu397

**Published:** 2014-12-21

**Authors:** Ian H. Cheeseman, Marina McDew-White, Aung Pyae Phyo, Kanlaya Sriprawat, François Nosten, Timothy J.C. Anderson

**Affiliations:** ^1^Texas Biomedical Research Institute, San Antonio, TX; ^2^Shoklo Malaria Research Unit, Mahidol-Oxford Tropical Medicine Research Unit, Faculty of Tropical Medicine, Mahidol University, Mae Sot, Thailand; ^3^Centre for Tropical Medicine, Nuffield Department of Medicine, University of Oxford, Oxford, United Kingdom

**Keywords:** drug resistance, malaria, pooled sequencing, rare variants

## Abstract

We explored the potential of pooled sequencing to swiftly and economically identify selective sweeps due to emerging artemisinin (ART) resistance in a South-East Asian malaria parasite population. ART resistance is defined by slow parasite clearance from the blood of ART-treated patients and mutations in the *kelch* gene (chr. 13) have been strongly implicated to play a role. We constructed triplicate pools of 70 slow-clearing (resistant) and 70 fast-clearing (sensitive) infections collected from the Thai–Myanmar border and sequenced these to high (∼150-fold) read depth. Allele frequency estimates from pools showed almost perfect correlation (Lin’s concordance = 0.98) with allele frequencies at 93 single nucleotide polymorphisms measured directly from individual infections, giving us confidence in the accuracy of this approach. By mapping genome-wide divergence (*F*_ST_) between pools of drug-resistant and drug-sensitive parasites, we identified two large (>150 kb) regions (on chrs. 13 and 14) and 17 smaller candidate genome regions. To identify individual genes within these genome regions, we resequenced an additional 38 parasite genomes (16 slow and 22 fast-clearing) and performed rare variant association tests. These confirmed *kelch* as a major molecular marker for ART resistance (*P* = 6.03 × 10^−6^). This two-tier approach is powerful because pooled sequencing rapidly narrows down genome regions of interest, while targeted rare variant association testing within these regions can pinpoint the genetic basis of resistance. We show that our approach is robust to recurrent mutation and the generation of soft selective sweeps, which are predicted to be common in pathogen populations with large effective population sizes, and may confound more traditional gene mapping approaches.

## Introduction

The spread of drug resistance alleles through malaria parasite populations has produced several key examples of evolution in action, including both hard and soft selective sweeps (Wootton et al. 2002; Nair et al. 2003, 2007). Drug resistance alleles compromising the efficacy of chloroquine, mefloquine, and sulphadoxine–pyrimethamine have arisen in South-East Asia and spread globally (Anderson and Roper 2005), resulting in the withdrawal of chloroquine and sulphadoxine–pyrimethamine as curative therapies. Drug resistance now threatens the great strides forward in reducing malaria burden made in the last decade, with emerging resistance to artemisinin (ART), the current global front-line therapy, of particular concern (Dondorp et al. 2010). ART resistance is measured as a reduction in the rate at which the parasite burden within a patient is reduced (the clearance rate [CR]). Clearance occurs rapidly in infections carrying sensitive parasites, with essentially all parasite biomass eradicated within 48 h (Dondorp et al. 2009), while in infections bearing resistant parasites it is common to detect parasites in the peripheral blood for up to 7 days, and treatment failure is common (Dondorp et al. 2009; Phyo et al. 2012; Carrara et al. 2013). ART resistance has been confirmed in multiple locations throughout South-East Asia, including Cambodia (Dondorp et al. 2009; Noedl et al. 2009) and Thailand (Phyo et al. 2012), and is suspected more broadly throughout the region (Ashley et al. 2014). Resistance is driven predominantly by parasite genetics (Anderson et al. 2010; Phyo et al. 2012; Takala-Harrison et al. 2013), with heritability estimates of 66% in Western Thailand (Nkhoma et al. 2013). A major determinant of ART resistance, the *kelch* gene (PF3D7_1343700), was recently identified (Ariey et al. 2014). The region in which this gene lies was previously identified as being under strong, positive selection through genome scans, and contains molecular markers with a significant association with parasite CR under treatment (Cheeseman et al. 2012; Miotto et al. 2013; Takala-Harrison et al. 2013; Ashley et al. 2014). It is currently unclear if mutations in this gene explain all the variation in CR between infections or if other genes are also involved in the parasite response to ART treatment.

Strong selection acting on a beneficial mutation drives both its allele frequency, and the allele frequencies of flanking neutral alleles (Smith and Haigh 1974; Barton 2000). This property of selective sweeps has been exploited to map genome regions with unusually high levels of divergence between populations (or phenotypic groups within a population) using *F*_ST_-like statistics (Holsinger and Weir 2009). *F*_ST_-based genome scans have been used to identify population specific selective sweeps in many organisms (i.e., [Akey et al. 2010; Simonson et al. 2010; Jones et al. 2012]) including malaria parasites (Cheeseman et al. 2012; Takala-Harrison et al. 2013). Such *F*_ST_ scans have been expanded to detecting regions of divergence within single populations by partitioning individuals by a given phenotype. These have been widely used, for instance, to map divergence between dwarf and normal lake whitefish (Hebert et al. 2013), parallel speciation in stick insects (Soria-Carrasco et al. 2014), the evolutionary history of rice (He et al. 2011), and to identify the targets of long-term selection experiments (Burke et al. 2010; Beissinger et al. 2014).

Allele frequencies can be accurately measured from deep sequencing of pooled populations. This has led to a great interest in using pooled sequencing to map regions of genomic divergence between populations (Boitard et al. 2012). Pooled sequencing can be performed rapidly and economically, and has been shown to produce highly accurate results in free living organisms (Neafsey et al. 2010; Turner et al. 2010; Bastide et al. 2013). However, there are specific challenges in applying pooled sequencing to malaria infections. First, there are methodological challenges because parasites are blood borne, and samples are frequently contaminated by human DNA, which can vary substantially between samples even after high-quality sample preparation (Venkatesan et al. 2012). Second, drug resistance mutations frequently have multiple origins (Musset et al. 2007; Nair et al. 2007, 2008) leading to “soft” sweeps (Pennings and Hermisson 2006). Soft sweeps are more challenging to detect than classical hard sweeps because the change in allele frequency of any one resistance lineage may be small.

This study was designed to evaluate the effectiveness and accuracy of pooled sequencing for malaria parasites obtained directly from infections. We show that a two-tier approach, which uses pooled sequencing followed by resequencing of a small number of parasite haplotypes and application of rare variant association tests, allows robust identification of a known causal gene. We also use coalescent simulation to examine thresholds for detection of selected genome regions, and highlight several other candidate genome regions.

## Results

### Validating Pooled Sequencing for Malaria Infections

**Fig. 1.**
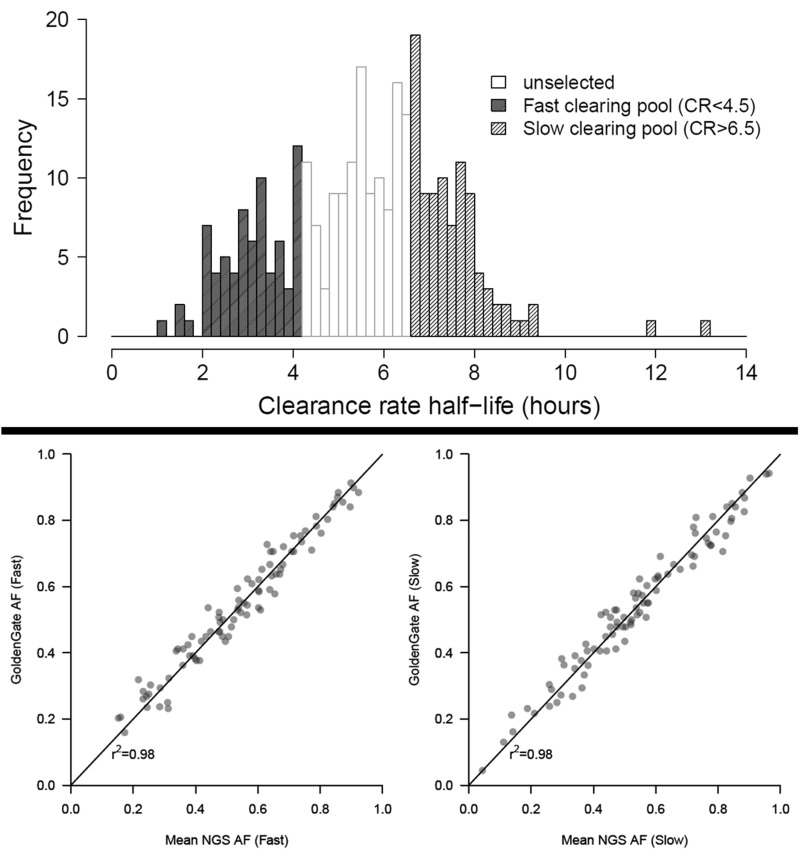
Pools of fast- and slow-clearing parasites accurately capture allele frequencies. (*A*) CR measurements from 291 infections in the 2011–2012 malaria season. Shading shows how individual infections were categorized for pooled sequencing. (*B* and *C*) Comparisons between the known allele frequency of pools (GoldenGate AF) and the allele frequency estimated from deep sequencing (mean NGS AF) for fast (*B*) and slow (*C*) clearing pools. The *r*^2^ values refer to Lin’s concordance.

To validate the use of pooled sequencing in identifying the determinants of antimalarial drug resistance, we measured parasite CR in 291 ART-treated patients presenting to clinics on the Thai–Burmese border in the 2011–2012 malaria season (fig. 1*A*). We classified these as slow (CR >6.5 h) or fast (CR <4.5 h) clearing, and genotyped the parasites from each infection using a custom 96- single nucleotide polymorphism (SNP) GoldenGate platform (Phyo et al. 2012). Thirty-eight (13%) infections contained greater than 1 multilocus parasite genotype; these were excluded leaving 253 single-clone infections. Forty-three infections contained perfect matches to other infections present in the data set (identical clones). Retaining all single clone infections in the analysis, including all representatives of identical clones, had little observable effect on allele frequencies (supplementary fig. S1, Supplementary Material online), and we opted to retain all single clones in the pools to maximize sample size. We selected the 70 fastest and 70 slowest clearing individuals (fig. 1*A*), and pooled equimolar concentrations of DNA from each. These six pools were sequenced in multiplex to approximately 150-fold coverage on a single lane of an Illumina HiSeq 2500. We generated allele frequency estimates from aligned read depth data, limiting our analysis to 23,292 high quality (Q30) SNPs with greater than 70-fold coverage across all pools after removing SNPs in large antigenic gene families. The 23,292 SNPs included in our analysis had a median per pool read depth of 154 (range: 70–250).

We explored how well our deep sequence data captured population allele frequencies. Using the genotypes of the 93 GoldenGate SNPs, we calculated the allele frequency of each SNP. We compared these to the mean allele frequency across replicate pools for the same sites and obtained highly concordant measures (Lin’s concordance 0.98, fig. 1*B* and *C*). This was marginally lower than expected from a binomial sampling of alleles based on their allele frequency alone (supplementary fig. S2, Supplementary Material online). We then measured the reproducibility of allele frequency estimation within pools across all 23,292 SNPs by performing pairwise comparisons between each replicate (Lin’s concordance mean = 0.975, range = 0.973–0.976, supplementary fig. S2, Supplementary Material online). This was substantially higher than concordance of allele frequency estimation between pools (supplementary fig. S2, Supplementary Material online). Finally, by estimating pairwise *F*_ST_ between technical replicates from within each pool, we were able to estimate the expected false positive rate due to technical artifacts. We detected 362/23,292 (1.55%) and 32/23,292 (1.47%) of SNPs with an *F*_ST_>0.1 within the fast and slow clearing pools, respectively. By calculating *F*_ST_ in 25 kb sliding windows with a 5 kb offset, we reduced the proportion of significant loci to 12/4,600 (0.26%) and 6/4,600 (0.13%) within fast and slow pools, respectively. This was further improved by excluding windows with high variability in estimating *F*_ST_ (standard error of the mean of between or within pool replicates >0.0125, windows containing <5 SNPs or >100 SNPs) after which no *F*_ST_ signals reached 0.1 within either fast or slow pools (supplementary fig. S3, Supplementary Material online). As we expect selective sweeps to drive allele frequencies of multiple linked SNPs simultaneously, calculating *F*_ST_ in windows should not affect our ability to detect strong, recent selective sweeps. We show below that this threshold (*F*_ST_>0.1) is appropriate using coalescent simulations, and that multiple SNPs within a 25 kb window are likely to be present at high *F*_ST_ where selection is acting.

### Scanning the Malaria Parasite Genome for ART Resistance Loci

**Fig. 2.**
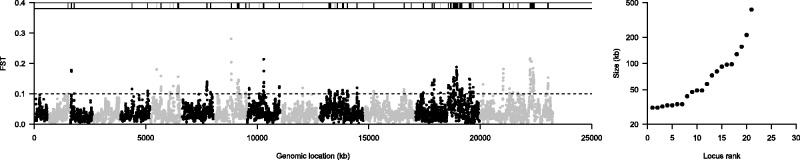
Genome-wide *F*_ST_ scan identifies known targets of ART resistance. (*A*). The *F*_ST_ score in 25 kb windows across the genome. The windows are arranged in order across the genome, with odd numbered chromosomes shown in black and evenly numbered chromosomes in gray. The *F*_ST_ threshold is shown by a dashed line and windows above this threshold shown in the bar at the top of the plot. Where multiple significant windows are detected within 100 kb of each other these regions are shown in black (listed in supplementary table S1, Supplementary Material online), whereas single windows are shown in gray. (*B*) The size of significant windows. Single windows are excluded from this plot.

We calculated *F*_ST_ in 25 kb windows for all 23,292 SNPs using the mean allele frequency of replicate pools of fast clearing and slow clearing pools of parasites. We identified 1,12/3,964 (2.8%) windows with an *F*_ST_>0.1 (fig. 2*A*). These windows were nonrandomly distributed across the genome, with 92 windows falling within 100 kb of another highly diverged window (*P* < 1 × 10^−^^5^, by permutation). After collapsing windows within 100 kb together, there were 19 multiwindow regions (supplementary table S1, Supplementary Material online). When ranking these by size (fig. 2*B*), the largest genome region (380 kb, chr. 13, 1,717,500–2,097,500) contains the recently identified ART resistance gene PF3D7_1343700 (Ariey et al. 2014) and has previously been shown to be under strong selection (Cheeseman et al. 2012; Miotto et al. 2013; Takala-Harrison et al. 2013). The second largest window (180 kb, chr 14, 2,252,500–2,432,500) is adjacent to two previously identified regions from independent studies comparing geographically separated ART-resistant and ART-sensitive parasite populations (103 kb, chr. 14, 2,433,083–2,536,420, (Takala-Harrison et al. 2013) and 125 kb, chr. 14, 2,730,000–2,855,000 [Cheeseman et al. 2012]).

### The Robustness of Pooled Sequencing to the Beneficial Mutation Rate

**Fig. 3.**
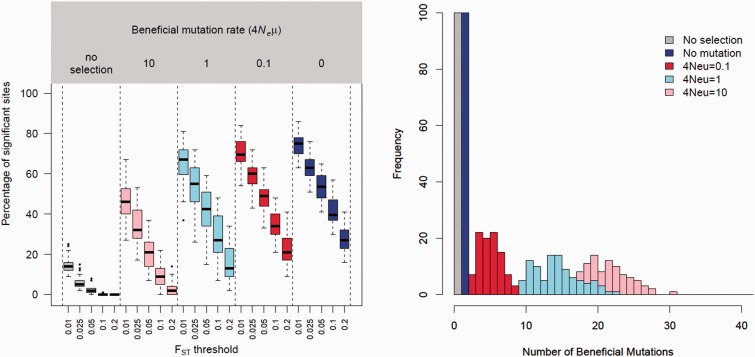
Pooled sequencing is robust to soft sweeps. (*A*) The results from coalescent simulations under varying beneficial mutation rates. The leftmost gray boxes show the results from neutral simulations in the absence of selection. The rightmost blue boxes show a hard sweep in the absence of further beneficial mutation. The three boxes in between (pink, light blue, and red bars) show simulations with increasing “hardness” as the population mutation rate (*4N_e_μL*) is reduced from 10 to 0.1. Each box summarizes the number of SNPs each simulation produced with an *F*_ST_ value above a given threshold (shown below the box). (*B*) The number of beneficial mutations observed during each simulation. One hundred simulations were run for each value of *4N_e_μL*.

To estimate whether our pooled sequencing approach is well powered to detect sweeps driven by strong selection in large parasite populations, and to establish appropriate empirical thresholds for identifying genome regions of interest, we performed coalescent simulations using msms (Ewing and Hermisson 2010). These simulations (outlined in supplementary fig. S4, Supplementary Material online) used parameters estimated from independent studies of malaria parasite populations. In settings such as the Thai–Burmese border the majority of malaria cases are treated with drugs, resulting in a strong selective pressure on the parasite population (Luxemburger et al. 1997). Consequently, drug resistance mutations are highly beneficial, with selection coefficients (*s*) on the order of 0.1 or higher (Nair et al. 2003). Similarly high selection coefficients have been observed in other treated parasite populations (Anderson and Roper 2005; Nwakanma et al. 2013), and the rise of ART resistance across the Thai–Burmese border over the last decade has been nearly linear and suggests *s* to be in the same order of magnitude for this drug (Phyo et al. 2012). We simulated a similarly sized population to our Thai–Burmese sample (*N_e_* = 10,000 [Joy et al. 2003]) where an adaptive mutation rises to 50% frequency with a trajectory described by *s*. We calculated *F*_ST_ between 70 individuals bearing a beneficial mutation and 70 without in a 25 kb region adjacent to, though not including, the selected locus. To estimate the background level of false positives expected we performed neutral simulations where no selection was included in the model and individuals were randomly assigned to two groups. In these simulations 10.4% of runs showed markers with an *F*_ST_ >0.1 (no selection, fig. 3*A*). This suggested a low, but not inconsequential false positive rate at this threshold. Notably, in these neutral simulations only a single marker in each run had an *F*_ST_ >0.1, these would be unlikely to produce significant hits in a windowed strategy and is in contrast to simulations with selection where greater than 20% of SNPs reached this threshold in plausible simulations (4*N_e_*µL = 0.1, fig. 3*A*). In our pooled sequencing experiments a *F*_ST_ threshold of 0.1 captures variants in the top 2.5% of values genome wide, providing further evidence that this threshold is reasonable.

Previously described drug resistance alleles in malaria parasites frequently have multiple origins (Musset et al. 2007; Nair et al. 2007, 2008). If a beneficial mutation has multiple origins this can lead to more complex patterns of variation flanking adaptive mutations than in a classical “hard” sweep (Smith and Haigh 1974), and may confound genome-wide scans for selection (Pritchard et al. 2010). To assess if recurrent mutation would impact our ability to identify a selective sweep in pooled data, we simulated the emergence of a beneficial allele in a population under a range of beneficial mutation rates (4*N_e_*µL = 0, 0.1, 1, and 10). For simulations of soft sweeps (4*N_e_*µL ≥0.1) we conditioned on the presence of ≥2 beneficial mutations, discarding simulations which did not reach this threshold. The actual number of beneficial mutations in each simulation is shown in figure 3*B*, with up to 30 beneficial mutations present in the most extreme simulations. At very soft sweeps (4*N_e_*µL = 10), 97.9% of our simulations contained markers with *F*_ST_ >0.1 between resistance classes (fig. 3*A*) with 100% of simulations containing high *F*_ST_ markers at lower, more plausible values of 4*N_e_*µL. The broad window of values for 4*N_e_*µL that we used for these simulations encompasses those plausible for *Plasmodium falciparum*. Obtaining a precise estimate of this compound parameter will be dependent on obtaining accurate per generation mutation rates and understanding the breadth of mutations which can result in the slow clearance phenotype.

### Implicating Single Genes within F_ST_ Peaks

**Fig. 4.**
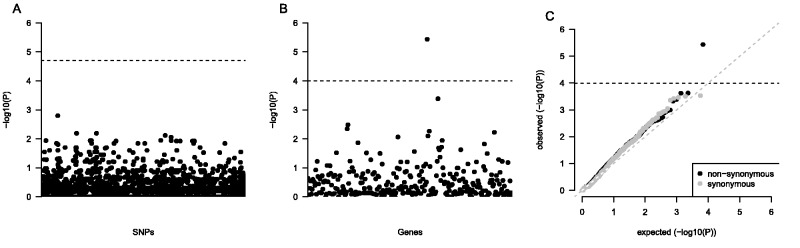
Association tests confirm a major ART resistance gene in sequenced parasites. Single marker association tests show no significant ART resistance markers (*A*). Burden tests show PF3D7_1343700 to be significantly associated with ART resistance (*B*). Quantile–quantile plot of burden test statistics from nonsynonymous (black) and synonymous (gray) SNPs (*C*). These suggest our burden test results were not influenced by *P*-value inflation.

Each of the 19 multiwindow peaks we detected contained multiple genes (supplementary table S1, Supplementary Material online). The total number of genes (493) is too large for direct functional characterization, and further prioritization is needed. We performed direct whole-genome sequencing of an independent set of 38 parasite genomes (16 fast clearing, 22 slow clearing) collected between 2008 and 2010 which were not present in either of the pools, and tested SNPs for association with CR. Traditional genome-wide association studies are based on simple population genetic models, whereby a region harbors a single causal mutation. We know these models are unlikely to hold in the case of *kelch* for which 17 causal mutations were recently described (Ariey et al. 2014). Furthermore, recurrent mutation and soft sweeps have been observed at several other antimalarial drug resistance genes (i.e., *pfmdr1* [Nair et al. 2007], *gch-1* [Nair et al. 2008], *mtDNA Co I* [Musset et al. 2007]). Single marker tests are therefore underpowered to detect associations where soft sweeps are likely. In keeping with this we fail to detect any statistical association between CR and SNPs residing within *F*_ST_ peaks for our 38 samples using single marker Fisher’s exact tests (fig. 4*A*).

**Fig. 5.**
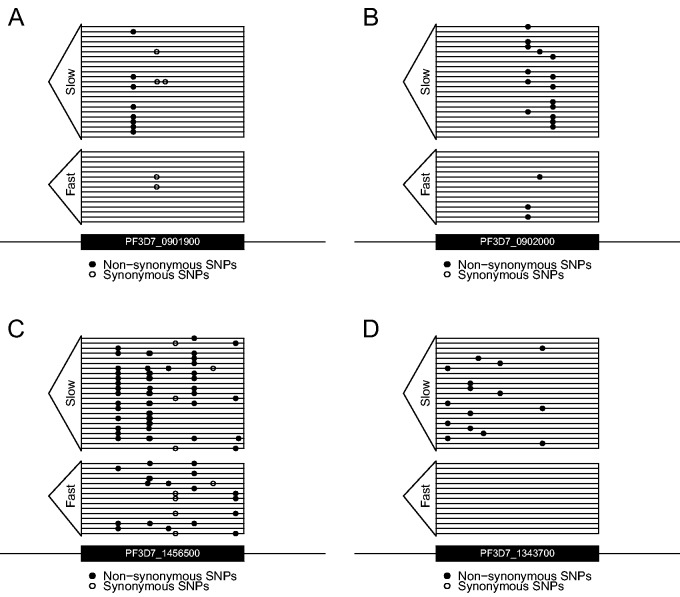
The pattern of variation in putative ART resistance markers. Direct whole-genome sequencing of 16 fast- and 22 slow-clearing parasites reveals a distinct pattern of variation in the *kelch* gene (*D*). Nonsynonymous SNPs (black circles) are only seen in the slow-clearing parasites, with 16/22 parasites containing a variant. Seven distinct alleles are seen in this small sample. No synonymous mutations were observed in either CR class. Variation in three other genes—PF3D7_0901900 (*A*), PF3D7_0902000 (*B*), PF3D7_1456500 (*C*)—which show an enrichment of derived, nonsynonymous mutations in slow-clearing parasite genomes though do not reach experiment-wide statistical significance.

We performed SKAT-O (Ionita-Laza et al. 2013) tests for each gene which fell within an *F*_ST_ peak. This test is one member of a powerful new class of statistics that performs group-wise association tests across sets of SNPs (i.e., genes) by aggregating the effects of rare and common variants (Li et al. 2013). These tests utilize all variants, but rare variants are upweighted. The *kelch* gene was directly implicated (*P* = 6.03 × 10^−^^6^), and was the only gene which reached experiment-wide statistical significance after Bonferroni correction (fig. 4*B*). To explore whether *P*-values from the SKAT-O tests show potential inflation, we calculated the genomic control statistic (*λ*; Devlin and Roeder 1999). No evidence of overinflation of *P*-values was evident (*λ* = 1.00). We also compared the distribution of *P*-values for SKAT-O tests using either nonsynonymous or synonymous mutations. Assuming synonymous mutations are neutral then no enrichment of mutations should be seen in drug resistance genes. Synonymous mutations yielded no evidence of an inflation of *P*-values (*λ* = 0.77), and no *P*-values neared significance and the distribution of *P*-values followed null expectations (fig. 4*C*). We see genes in several other selected regions that show association, but do not reach experiment-wide statistical significance after Bonferroni correction (fig. 5). These include: PF3D7_0901900 (*P* = 0.0048), PF3D7_0902000 (*P* = 0.0039), and PF3D7_1456500 (*P* = 0.0072). To ensure we had not excluded potentially important genes from our analysis by only including genes within *F*_ST_ peaks we reran the SKAT-O analysis genome wide. Following correction for multiple testing no genes aside from kelch were associated with slow clearance (supplementary fig. S5, Supplementary Material online).

## Discussion

ART resistance has spread rapidly on the Thai–Burmese border, and effective genomic epidemiology tools which can rapidly identify causal markers are in much need. To this end pooled sequencing has several major advantages. It is amenable to mapping strong, recent selection and is robust to soft sweeps, which are widely seen surrounding drug resistance alleles (Messer and Petrov 2013), including several antimalarials such as atovaquone (Musset et al. 2007), mefloquine (Triglia et al. 1991; Nair et al. 2007), and GTP-cyclohydrolase I (Nair et al. 2008). Pooled sequencing can be applied using simple experimental designs. We accurately mapped resistance to a single gene using pooled sequencing and small scale resequencing in a single population, using a total of 178 individual parasites (140 for pooled sequencing and 38 for resequencing). Pooled sequencing is also highly economical. Our pooled sequencing cost approximately $1,200, including library preparation (labor costs not included). To individually sequence all 140 genomes from the pooling experiment at 2013, prices would cost approximately $35,000 and would require substantial additional preparation and analysis time.

### Mapping Selective Sweeps Driven by ART Resistance

We have two lines of evidence which give us confidence that the high *F*_ST_ genome regions we detected are due to the emergence of ART resistance. First, we expect a low level of technical variation to produce false positives. We observed excellent agreement between the known allele frequencies determined from GoldenGate genotyping and the estimated allele frequencies determined from pooled sequencing; and excellent agreement of allele frequency estimations between replicates from the same condition (fast/slow clearing). Self–self comparisons of *F*_ST_ in windows across the genome produced few windows with high *F*_ST_. These self–self comparisons are conservative compared with our between-pool genome-scan, in each case the allele frequency from a single fast or slow clearing replicate was used as a comparator, while in our between-pool genome-scan we took the mean allele frequency across replicates to estimate *F*_ST_. We also detect a far higher number of significant windows in our between-pool genome-scan (112) than in our within-pool genome-scans (0). Second, we have performed coalescent simulations to estimate the level of divergence expected between fast- and slow-clearing parasites under a range of beneficial mutation rates. These simulations suggest that for our specific evolutionary scenario, the emergence and rapid spread of a highly beneficial allele in a well mixed population, we should expect regions under selection to show quite distinct levels of divergence from those expected under neutrality (fig. 3*A*). Notably, we expect to be able to clearly detect divergence using pooled sequencing in the presence of realistic beneficial mutation rates, including those which would lead to soft sweeps. We used a threshold based on neutral simulations, rather than on *F*_ST_ comparisons based on technical replicates, in order to account for additional allele frequency variation expected from 1) genetic drift within drug sensitive and resistance subpopulations of parasites and 2) from sampling of individuals from within these populations.

The success of *F*_ST_ mapping in the presence of soft sweeps in this work is likely due to the setting in which we applied pooled sequencing. We applied pooled sequencing to a particularly clear example of natural selection. On the Thai–Burmese border selection for resistance is very strong (s≈0.1), very uniform (most patients with malaria are treated), and very recent (ART has only been widely used since 1995 (Carrara et al. 2013), 70–140 parasite generations ago). The observed background *F*_ST_ between slow- and fast-clearing parasites drawn from the same population is considerably lower than observed between geographically separated populations from the same region. For example, we previously compared parasite populations showing distinctive parasite clearance patterns from neighboring South-East Asian countries (Thailand, Laos, and Cambodia). The mean *F*_ST_ between countries of 0.108 (Cheeseman et al. 2012) was three times greater than we observed in this study (*F*_ST_ = 0.036). The ability to identify soft sweeps from pooled sequencing may not generalize well to other settings where selection is more ancient, weaker or from standing variation (Hermisson and Pennings 2005; Pennings and Hermisson 2006; Pritchard et al. 2010) or where there is a more substantial level of background noise generated by divergence between pools. It would be advisable to perform in silico simulations as we have done here prior to embarking on any more complex system.

There were additional technical reasons for the success of our pooled sequencing approach. We placed special emphasis on depleting human DNA contamination from our samples prior to pooling by filtering RBCs through CF11 columns to remove human white blood cells prior to DNA extraction (Venkatesan et al. 2012). These have been shown to efficiently remove white blood cells from whole blood. CF11 columns are not limited to use in a well equipped laboratory, as we have done here, and can be applied directly in a field setting (Venkatesan et al. 2012). In addition to high-quality preparation of DNA, we selected patients who were hyperparasitaemic (see Materials and Methods). These patients have a parasitaemia of greater than 4%, so a higher proportion of parasite DNA is present in blood samples. It has been previously suggested that pooled sequencing can capture the allele frequency spectra of a population with an accuracy approaching sequencing individuals, provided the depth of sequencing is high enough (Futschik and Schlotterer 2010). Detailed modeling of pooled sequencing experiments with a comparable size to that presented here (100 individuals pooled [*N*] and sequenced to 100× read depth) have shown that above an allele frequency of 5/*N* there is negligible bias in allele estimation (Lynch et al. 2014).

### Identification of a Major ART Resistance Gene

We were able to implicate a single ART resistance gene using small scale resequencing of 38 samples. This gene, *kelch* (Ariey et al. 2014), is strongly implicated as a key molecular marker of ART resistance from epidemiological data and parasites engineered to carry the C580Y mutation show a significant reduction in drug sensitivity (Ghorbal et al. 2014). We provide strong support for a major role in resistance using rare variant association test statistics. Our power to confirm *kelch* as a major ART-resistance marker is not limited to SKAT-O. We performed 26 other rare variant tests on PF3D7_1343700 (supplementary table S2, Supplementary Material online) and found all gave a significant result at *P* < 0.05 and 16/26 produced genome-wide significant *P*-values.

Rare variant association tests (including burden tests and nonburden tests such as SKAT-O) are particularly well suited for identifying causal genes subject to recurrent mutation. The *kelch* gene provides a showcase example of a situation where these tests are more appropriate than single marker tests. Here seven, independent nonsynonymous, derived, mutations (E252Q, P441L, M476I, R515K, G538V, P574L, A675D) targeting the *kelch* gene are present at frequencies ranging from 4% to 18% in resistant parasites and are completely absent from sensitive ones (fig. 5). We also observed five mutations in the slow clearing pools of parasites (P441L (21%), L457F (10%), R561H (28%), C580Y (25%), A675D (11%)). We did not observe synonymous mutations in the *kelch* gene in any parasites examined. Six of these mutations (underlined above) were not observed in ART-resistant Cambodian parasite populations (Ariey et al. 2014). Six of the 22 (27%) slow-clearing parasites were fully sequenced but lacked variants in this gene. Other mutations not directly assessed by our rare variant association tests such indels, copy number variants, or noncoding variants may modulate gene function in these six parasites. Alternatively, genes elsewhere in the parasite genome may contribute to slow clearance. Given the patterns of diversity we see in the kelch gene, loss-of-function (LOF) is a plausible resistance mechanism, because there are multiple ways to inactivate a gene. Soft sweeps are also especially likely where resistance results from through LOF mutations (Pennings and Hermisson 2006; Messer and Petrov 2013), because many different mutations can disrupt gene function. However, the absence of stop codons observed in kelch, and the variation in CR for different kelch mutations (Ariey et al. 2014; Ashley et al. 2014) suggests a reduction or alteration in enzyme function may be operating in this case.

There have a number of recent studies which have explored patterns of recent, positive selection in ART-resistant parasite populations (Cheeseman et al. 2012; Miotto et al. 2013; Takala-Harrison et al. 2013). Each of these studies identified a list of genes or regions of interest which may play a role in ART resistance, generally through genome scans for positive selection. Each of these studies shows disparity in the genes identified as putative resistance genes, and only two (including this study) include *kelch* as a candidate gene (supplementary fig. S6, Supplementary Material online). However, the region of chr. 13 surrounding this gene shows strong signatures of selection in all studies, with eight of the ten genes identified by at least four of five genome scans residing in this area, including PF3D7_1343800 which is directly adjacent to the *kelch* gene. In this study, we identified additional novel loci that have not previously been associated with ART resistance. For example, 330 of the genes within our selected *F*_ST_ regions were in regions not previously identified through genome-wide scans for selection.

We performed two independent analyses to first identify regions of the genome under selection and then implicate a single gene as driving resistance in our largest selective sweep. We believe such a two step analysis hold several advantages. Most GWAS studies are agnostic to which genes may control a phenotype, and therefore analyze all available SNPs, which compromises statistical power. Where we have prior support for a region this information should be used to increase the power of association analysis. By limiting our interest to approximately 500 genes we reduce the multiple test correction by 13.5-fold. Where large sample sizes are difficult to obtain this may prove to be a particularly useful strategy. In our data set, this increase in power does result in additional loci being detected. Furthermore, a genome-wide scan of all genes using SKAT-O confirms we had not excluded genes which would have reached genome-wide significance (supplementary fig. S5, Supplementary Material online). A modest increase in sample size of individual sequences may resolve additional loci, notably on chromosome 14, the second largest peak in our pooled sequencing.

Analysis of divergence between pools alone is not sufficient to implicate individual genes. This was apparent when we reanalyzed our pools either gene-by-gene or SNP-by-SNP (supplementary fig. S6, Supplementary Material online). The gene-by-gene analysis shows kelch to be above our threshold (*F*_ST_ =0.16), though multiple adjacent genes in the same region show higher *F*_ST_. Similarly, kelch did not harbor the most diverged single SNP. When we obtain single genome sequence data it is clear why we see this pattern. There are multiple, low frequency SNPs in kelch (fig. 5*D*) which are absent in fast-clearing parasites. Each of these alone only has a modest *F*_ST_.

By ranking the high *F*_ST_ genome regions by size we make the implicit assumption that size is an indicator of the importance of a region. Although this may in general be the case (for instance the largest region we detect contains kelch, a major effect mutation) there are several scenarios which may result in large effect mutations producing smaller genome regions with evidence of selection (Smith and Haigh 1974; Charlesworth et al. 1997; Feder and Nosil 2010).

Classical methods for conducting genome-wide association studies that assume a single causative mutation may fail to detect loci where recurrent mutation underlies the phenotype of interest (Thornton et al. 2013). Combining pooled sequencing for localization of genome regions of interest, followed by the application of tests such as SKAT-O, which are permissive to differing models of trait evolution, may represent a fruitful direction for unraveling these complex scenarios. These approaches may be particularly important for malaria and other pathogens, where both theoretical prediction and empirical observation suggest that multiple origins of drug resistance are common (Chen et al. 2007; Musset et al. 2007; Nair et al. 2007; Pennings 2012; Messer and Petrov 2013).

## Materials and Methods

### Collection of Parasites

Blood samples were collected from *P. falciparum*-infected patients with uncomplicated hyperparasitaemia (defined as ≥4% of red cells parasitized without clinical evidence of severe malaria) admitted to four malaria clinics spanning a 150 km region of the North-Western border of Thailand (Cheeseman et al. 2012; Phyo et al. 2012). The majority of patients came from adjacent Burma. Treatment was with a 7-day regimen of oral artesunate (4 mg/kg initially then 2 mg/kg once daily for 7 days) usually combined with either mefloquine (25 mg/kg in two divided doses), doxycycline (4 mg/kg/day for 7 days), or clindamycin (5 mg/kg three times daily for 7 days).

### Measurement of Parasite Clearance Half-Life

Parasite clearance half-lives were measured following (Phyo et al. 2012). Following treatment with ART combinations, blood smears were made every 6 h until patients were slide negative and parasite counts were read per 1,000 red cells (thin film) or 500 white cells (thick film). In brief, we fitted the decay in parasitaemia using a standardized fitting procedure (Flegg et al. 2011) which separates the variable initial lag-phase from the subsequent log-linear decline. We expressed the slope of the log-linear phase as the parasite clearance half-life (*t*_½_ *P*) which is the time required for parasitaemia to fall by half.

### Generation of Pooled Samples

Leukocytes were depleted from blood samples using CF11 columns (Whatmann) to minimize the level of human DNA contamination (Venkatesan et al 2012). DNA was then extracted from the RBC fractions of infected patient blood using the Gentra PureGene kit (QIAGEN) and DNA concentration was quantified using a Qubit fluorometer (Invitrogen). Initial genotyping was performed using a custom Illumina GoldenGate SNP typing assay (design described in Phyo et al. (2012). We followed the manufacturer’s instructions for genotyping except for the annealing stage which we extended from 2 to 16 h, to account for the high AT content of the *P. falciparum* genome (Gardner et al. 2002). Samples were considered to contain multiple genotypes infections if greater than 5 SNPs showed heterozygous base calls. Multiple genotype infections were excluded.

We pooled equimolar amounts of the fastest clearing 70 parasites (CR <4.5 h) and slowest clearing 70 parasites (CR >6.5 h) from the blood samples collected in 2011–2012. These pools were constructed in triplicate with each replicate used for a single sequencing library. An additional set of 38 individual parasite genotypes (22 slow clearing [CR >5 h] and 16 fast clearing [CR <2.5 h]) collected in 2008–2010 were sequenced directly. Library preparations were performed identically for pooled and nonpooled samples.

### Genome Sequencing

Two micrograms of DNA was sheared using a Covaris S-series sonicator (Covaris; duty cycle 20%, time 180 s, intensity 5, cycle burst 200, power 37 W, temperature 7 °C, mode freq sweeping). Sheared DNA was end-repaired, A-tailed and multiplex-indexed adaptors ligated using NEBnext library preparation kits for Illumina (New England Biolabs). Following (Quail et al. 2011; Oyola et al. 2012) we replaced the DNA polymerase with Kapa HiFi (Kapa Biosystems) and used Agencourt AMPure XP beads (Beckman Coulter) for sample purification. The Kapa SYBR Fast ABI Prism qPCR kit (Kapa Biosystems) was used to quantify templates before they were multiplexed (12 samples/lane) and sequenced on an Illumina HiSeq 2000. Raw Sequence data were demultiplexed and. fastq files generated using CASAVA 3.0 before further analysis.

One hundred one base pairs paired-end reads from.fastq files were mapped against the *P. falciparum* genome reference strain 3D7 v9.2 (http://plasmodb.org/common/downloads/release-9.2/Pfalciparum3D7/fasta/data/) using BWA v0.6.1 (Li and Durbin 2009). The resulting BAM files were cleaned to remove reads which map off chromosomes and polymerase chain reaction duplicates removed using picard v1.56 (http://picard.sourceforge.net/). The Genome Analysis Toolkit v2.3-9 (DePristo et al. 2011) was used to realign around indels and generate/recalibrate base quality scores before final SNP calling was performed using the UnifiedGenotyper. Variant quality scores were then recalibrated and variants removed if they failed any of the following quality metrics (QUAL <100.0, FS < 50, BaseQRankSum −2>X>2, MQRankSum −2>X> 2, QD < 10). For *P. falciparum* analysis well annotated SNPs were used for variant quality score recalibration (http://plasmodb.org/plasmo/). For nonpooled samples we additionally recalled SNPs using the GATK HaplotypeCaller from v2.8-1 of the Genome Analysis Toolkit after restricting calling to genome regions with significant *F*_ST_ hits.

### Statistical Approaches

We measured the correlation between known and estimated allele frequencies using Lin’s Concordance (Lin 1989) in the epiR package (http://cran.r-project.org/web/packages/epiR/index.html). This was used in preference to more commonly used correlation coefficients as this measures the fit to a line with intercept 0, slope 1 (the diagonal in fig. 1*B* and *C*). We performed random sampling of allele frequencies to asses if our correlations were as expected given random sampling of alleles from a pool using the approaches described in Zhu et al. (2012). *F*_ST_ was estimated using PoPoolation2 (Kofler et al. 2011) using the settings window size = 1, step size = 1,–karlsson for single marker estimates and window size = 25,000, step size = 5,000, -karlsson for sliding window estimates.

Simulations of a selective sweep (described in supplementary fig. S4, Supplementary Material online) were performed using msms (Ewing and Hermisson 2010) to examine the expected magnitude and pattern of allele frequency divergence within parasite populations under recent strong drug selection. We used the following parameters: -s 100 -N 10000 -ms 140 100 -r 10 -SAA *2N_e_s* -SAa *N_e_s* -Smark -Sp 0 -SF 0.000000075 1 0.5 -Smu *4N_e_*µL where *2N_e_s* is the product of the effective population size and the selection coefficient and *4N_e_*µL is the product of the effective population size, the beneficial mutation rate, and the mutational target size. Under this strategy we simulate the rise of drug resistance where a single gene underlies resistance with a heritability of 1. *F*_ST_ from these simulations was estimated using the hierfstat program (Goudet 2005).

Rare variant association tests were performed in SKAT (Ionita-Laza et al. 2013) using the “optimal.adj” method and a linear kernel. Nonsynonymous and synonymous variants were identified using snpEff v3.3 (Cingolani et al. 2012). We estimated the ancestral allele of each mutation in nonpooled genome sequences by aligning short read data from a *P. reichenowi* sequencing experiment (Otto et al. 2014; http://www.sanger.ac.uk/resources/downloads/protozoa/plasmodium-reichenowi.html, ERR019329-ERR019336) to the 3D7 genome. In sites where it was not possible to identify the ancestral allele due to missing or mixed sequencing data we used the reference strain allele. As the reference strain, 3D7, was isolated prior to widespread ART use, and is likely of African origin, drug resistance mutations are likely to be nonreference. Malaria parasites are haploid, though there are frequently mixed base calls in next-generation sequence data, arising from either technical bias or where two distinct parasite genotypes are present in a sample. Although we restricted our sequencing to single clone isolates a number of mixed base calls were observed. At these sites we adopted a dominant model where the presence of a nonreference SNP at a heterozygous site was treated as a homozygous nonreference SNP. As the power of rare variant tests is compromised by retaining neutral alleles in the test we removed all SNPs where the derived allele was not present in the slow-clearing parasites or had a derived allele frequency greater than 50% in the fast-clearing parasites.

### Data Access

Raw sequence data have been submitted to the NCBI Sequence Read Archive (SRA; http://www.ncbi.nlm.nih.gov/sra/) under project numbers PRJNA260844 and PRJNA260845.

## Supplementary Material

Supplementary figures S1–S7 and tables S1 and S2 are available at *Molecular Biology and Evolution* online (http://www.mbe.oxfordjournals.org/).

Supplementary Data
