# Supplementary material for: Pooled Sequencing and Rare Variant Association Tests for Identifying the Determinants of Emerging Drug Resistance in Malaria Parasites
Source: Mol Biol Evol. 2014 Dec 21;32(4):1080–90. doi: 10.1093/molbev/msu397 (PMC4379400; doi:10.1093/molbev/msu397)
Supplement: Supplementary Data [file supp_32_4_1080__index.html]

Pooled Sequencing and Rare Variant Association Tests for Identifying the Determinants of Emerging Drug Resistance in Malaria Parasites — Pooled Sequencing and Rare Variant Association Tests for Identifying the Determinants of Emerging Drug Resistance in Malaria Parasites — Supplementary Data 

# Pooled Sequencing and Rare Variant Association Tests for Identifying the Determinants of Emerging Drug Resistance in Malaria Parasites

## Supplementary Data

files

**Files in this Data Supplement:**

- Supplementary Data - pptx file
- Supplementary Data - xlsx file
- Supplementary Data - xlsx file
