# Supplementary material for: Pooled Sequencing and Rare Variant Association Tests for Identifying the Determinants of Emerging Drug Resistance in Malaria Parasites
Source: Mol Biol Evol. 2014 Dec 21;32(4):1080–90. doi: 10.1093/molbev/msu397 (PMC4379400; doi:10.1093/molbev/msu397)
Supplement: Supplementary Data [file supp_msu397_Supplementary_Figures.pptx]

## Slide 1
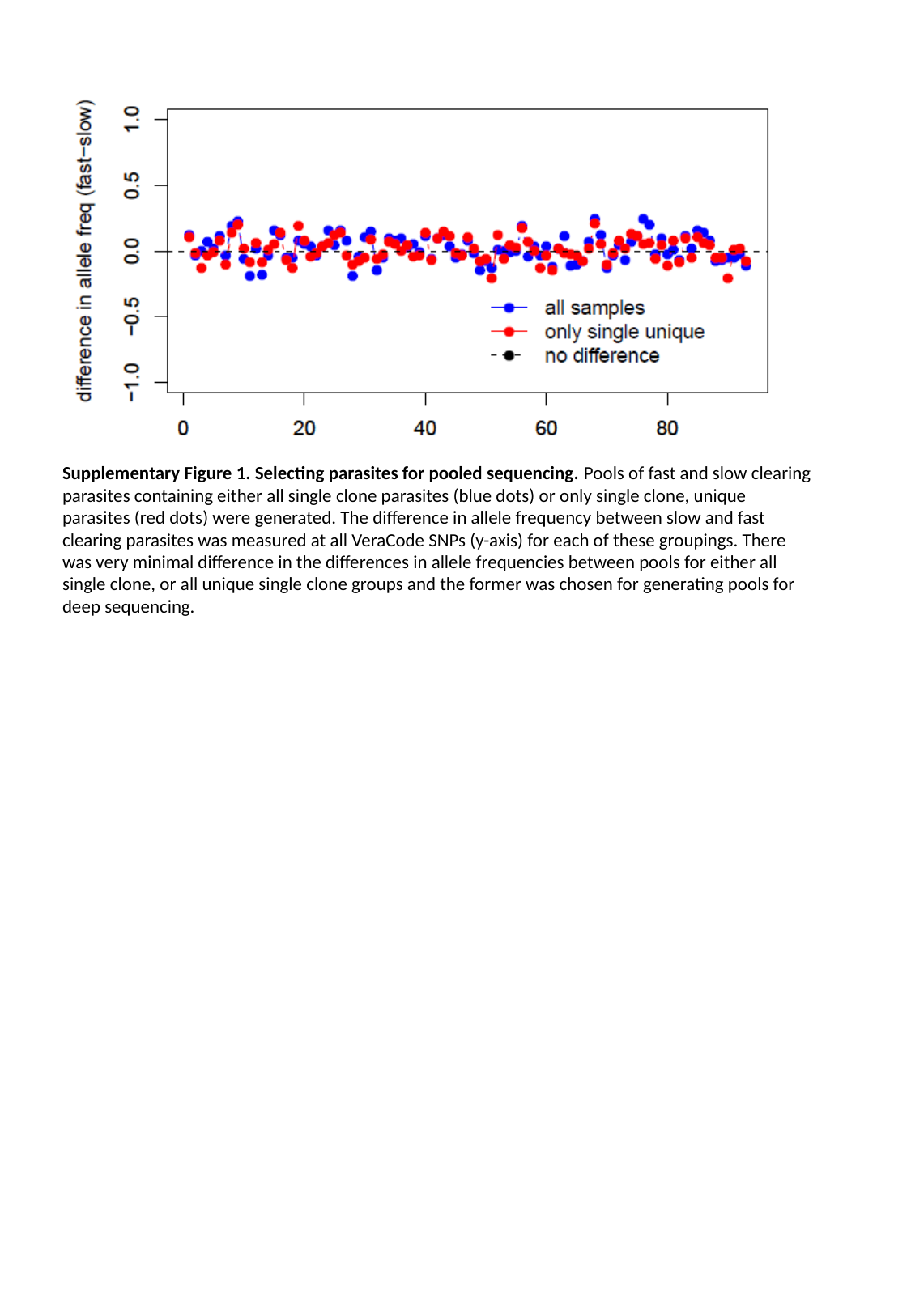

Supplementary Figure 1. Selecting parasites for pooled sequencing. Pools of fast and slow clearing parasites containing either all single clone parasites (blue dots) or only single clone, unique parasites (red dots) were generated. The difference in allele frequency between slow and fast clearing parasites was measured at all VeraCode SNPs (y-axis) for each of these groupings. There was very minimal difference in the differences in allele frequencies between pools for either all single clone, or all unique single clone groups and the former was chosen for generating pools for deep sequencing.

## Slide 2
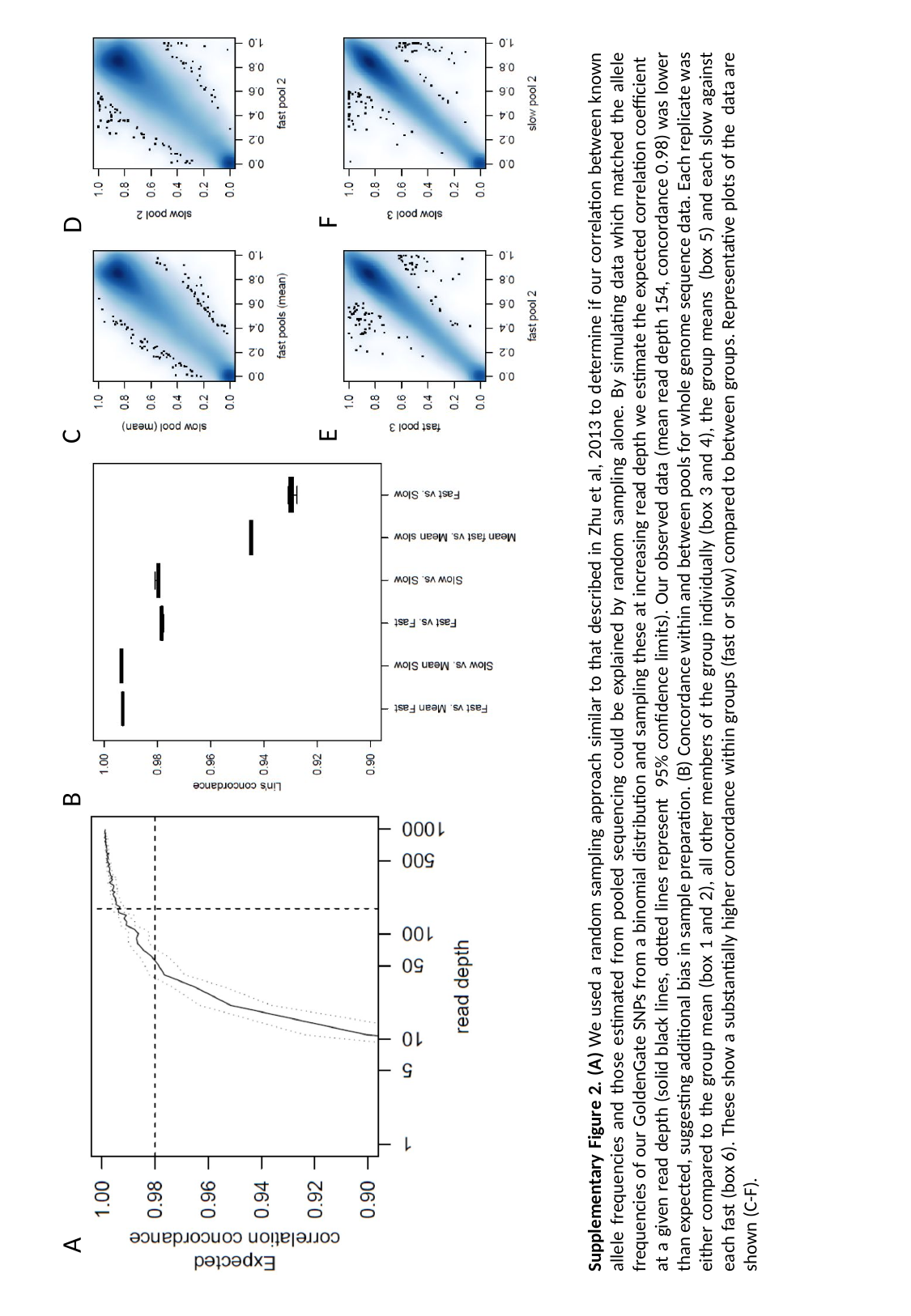

F
D
E
C
Supplementary Figure 2. (A) We used a random sampling approach similar to that described in Zhu et al, 2013 to determine if our correlation between known allele frequencies and those estimated from pooled sequencing could be explained by random sampling alone. By simulating data which matched the allele frequencies of our GoldenGate SNPs from a binomial distribution and sampling these at increasing read depth we estimate the expected correlation coefficient at a given read depth (solid black lines, dotted lines represent 95% confidence limits). Our observed data (mean read depth 154, concordance 0.98) was lower than expected, suggesting additional bias in sample preparation. (B) Concordance within and between pools for whole genome sequence data. Each replicate was either compared to the group mean (box 1 and 2), all other members of the group individually (box 3 and 4), the group means (box 5) and each slow against each fast (box 6). These show a substantially higher concordance within groups (fast or slow) compared to between groups. Representative plots of the data are shown (C-F).
B
A

## Slide 3
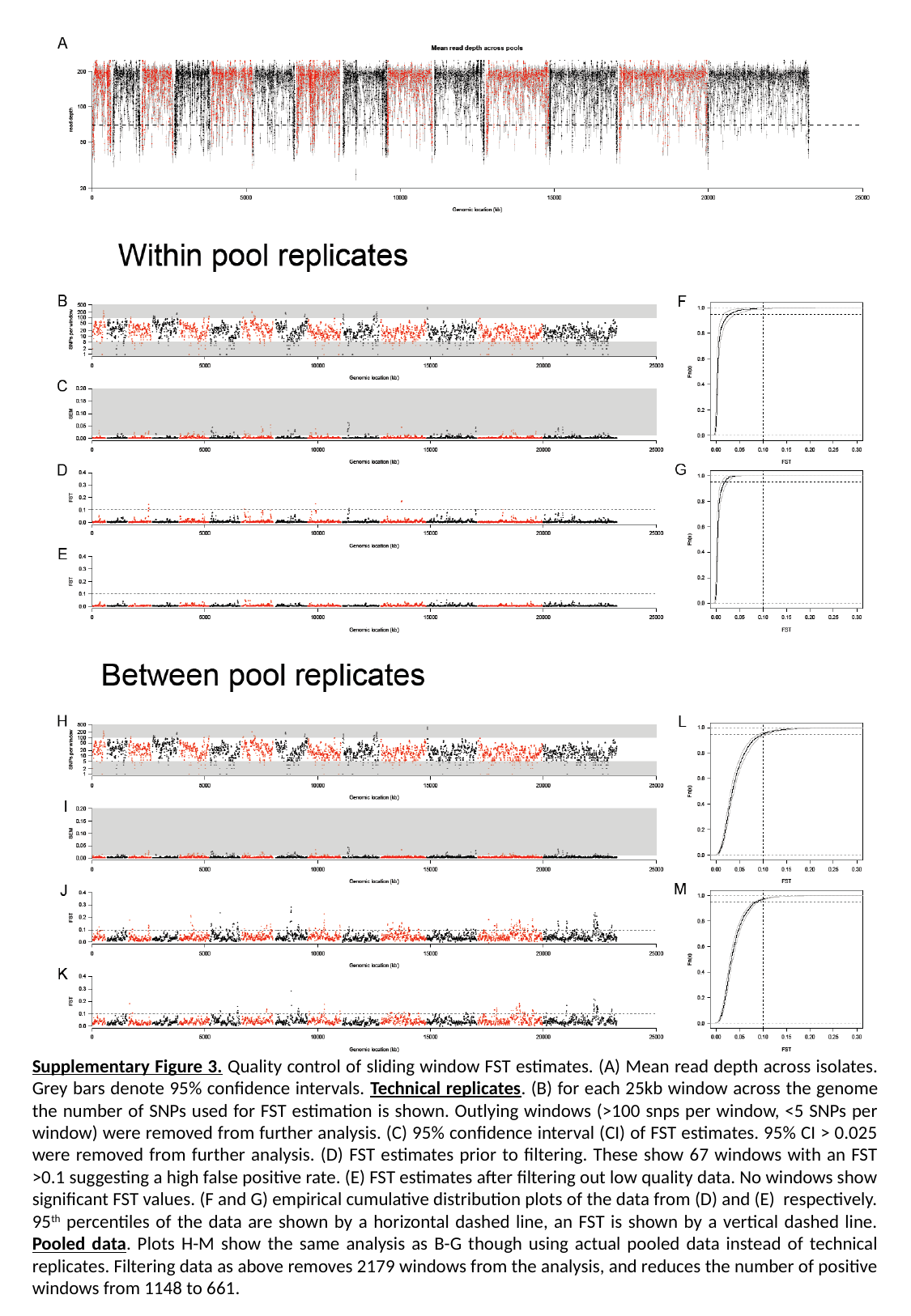

Supplementary Figure 3. Quality control of sliding window FST estimates. (A) Mean read depth across isolates. Grey bars denote 95% confidence intervals. Technical replicates. (B) for each 25kb window across the genome the number of SNPs used for FST estimation is shown. Outlying windows (>100 snps per window, <5 SNPs per window) were removed from further analysis. (C) 95% confidence interval (CI) of FST estimates. 95% CI > 0.025 were removed from further analysis. (D) FST estimates prior to filtering. These show 67 windows with an FST >0.1 suggesting a high false positive rate. (E) FST estimates after filtering out low quality data. No windows show significant FST values. (F and G) empirical cumulative distribution plots of the data from (D) and (E) respectively. 95th percentiles of the data are shown by a horizontal dashed line, an FST is shown by a vertical dashed line. Pooled data. Plots H-M show the same analysis as B-G though using actual pooled data instead of technical replicates. Filtering data as above removes 2179 windows from the analysis, and reduces the number of positive windows from 1148 to 661.

## Slide 4
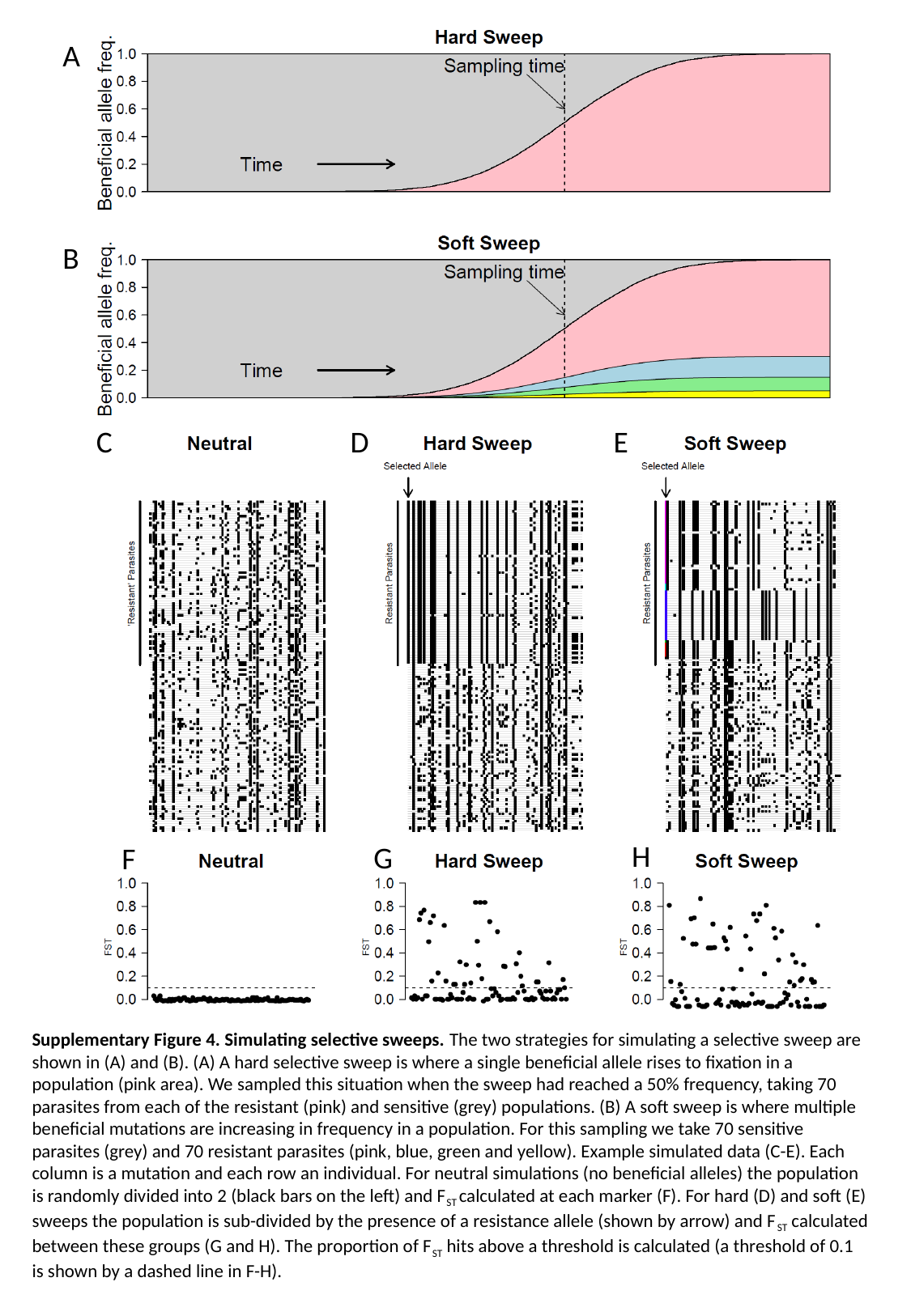

A
B
C
D
E
H
G
F
Supplementary Figure 4. Simulating selective sweeps. The two strategies for simulating a selective sweep are shown in (A) and (B). (A) A hard selective sweep is where a single beneficial allele rises to fixation in a population (pink area). We sampled this situation when the sweep had reached a 50% frequency, taking 70 parasites from each of the resistant (pink) and sensitive (grey) populations. (B) A soft sweep is where multiple beneficial mutations are increasing in frequency in a population. For this sampling we take 70 sensitive parasites (grey) and 70 resistant parasites (pink, blue, green and yellow). Example simulated data (C-E). Each column is a mutation and each row an individual. For neutral simulations (no beneficial alleles) the population is randomly divided into 2 (black bars on the left) and FST calculated at each marker (F). For hard (D) and soft (E) sweeps the population is sub-divided by the presence of a resistance allele (shown by arrow) and FST calculated between these groups (G and H). The proportion of FST hits above a threshold is calculated (a threshold of 0.1 is shown by a dashed line in F-H).

## Slide 5
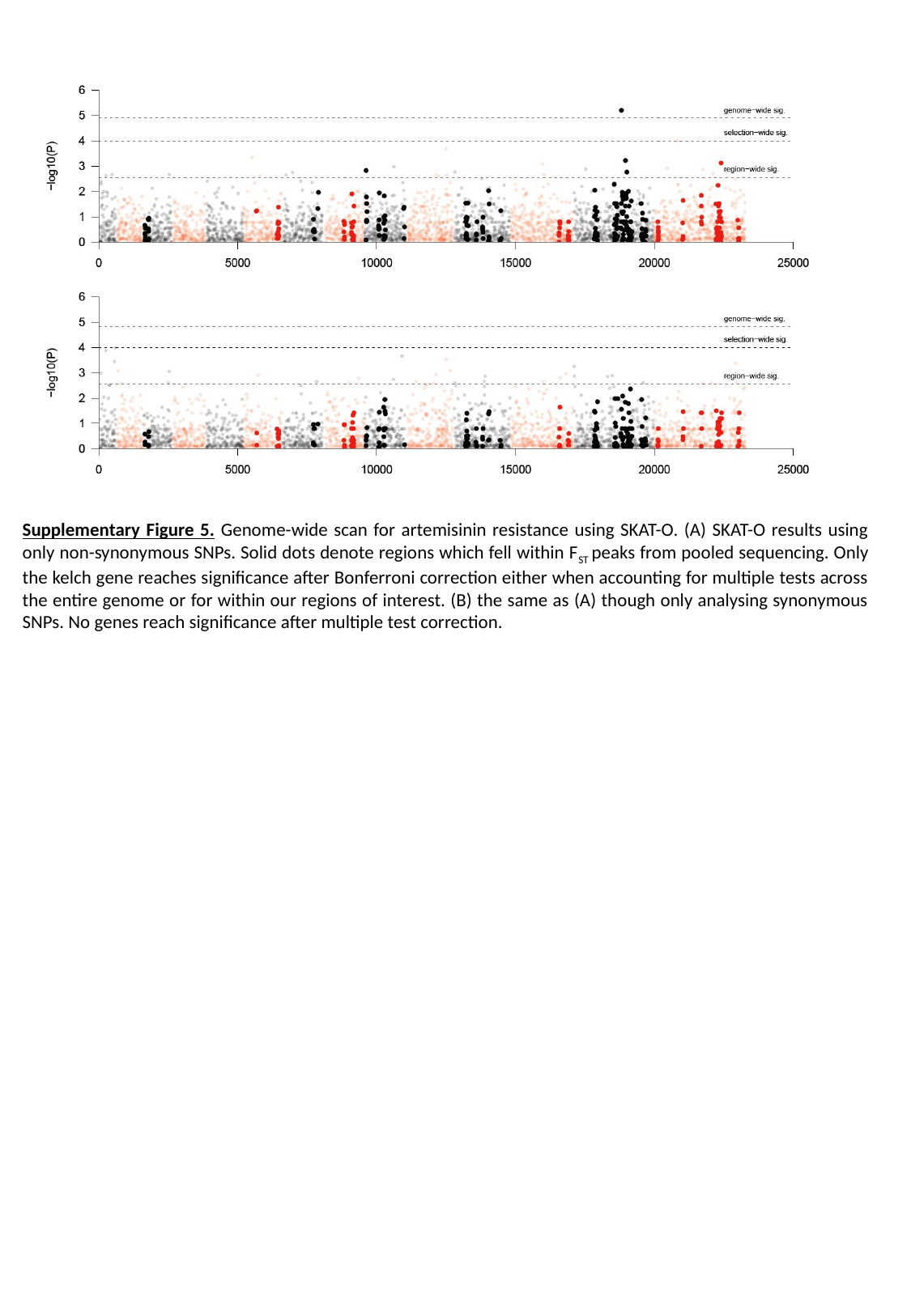

Supplementary Figure 5. Genome-wide scan for artemisinin resistance using SKAT-O. (A) SKAT-O results using only non-synonymous SNPs. Solid dots denote regions which fell within FST peaks from pooled sequencing. Only the kelch gene reaches significance after Bonferroni correction either when accounting for multiple tests across the entire genome or for within our regions of interest. (B) the same as (A) though only analysing synonymous SNPs. No genes reach significance after multiple test correction.

## Slide 6
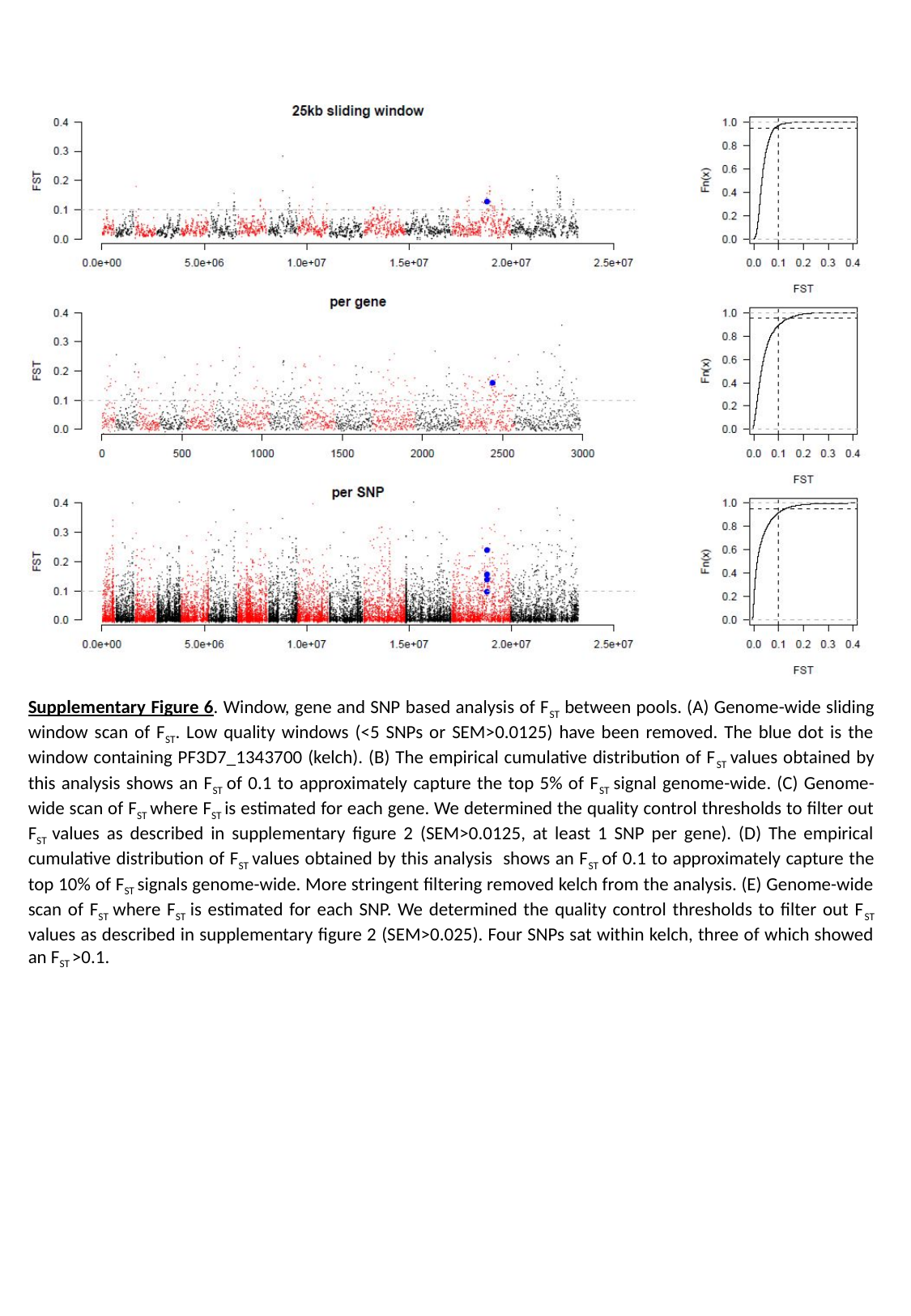

Supplementary Figure 6. Window, gene and SNP based analysis of FST between pools. (A) Genome-wide sliding window scan of FST. Low quality windows (<5 SNPs or SEM>0.0125) have been removed. The blue dot is the window containing PF3D7_1343700 (kelch). (B) The empirical cumulative distribution of FST values obtained by this analysis shows an FST of 0.1 to approximately capture the top 5% of FST signal genome-wide. (C) Genome-wide scan of FST where FST is estimated for each gene. We determined the quality control thresholds to filter out FST values as described in supplementary figure 2 (SEM>0.0125, at least 1 SNP per gene). (D) The empirical cumulative distribution of FST values obtained by this analysis shows an FST of 0.1 to approximately capture the top 10% of FST signals genome-wide. More stringent filtering removed kelch from the analysis. (E) Genome-wide scan of FST where FST is estimated for each SNP. We determined the quality control thresholds to filter out FST values as described in supplementary figure 2 (SEM>0.025). Four SNPs sat within kelch, three of which showed an FST >0.1.

## Slide 7
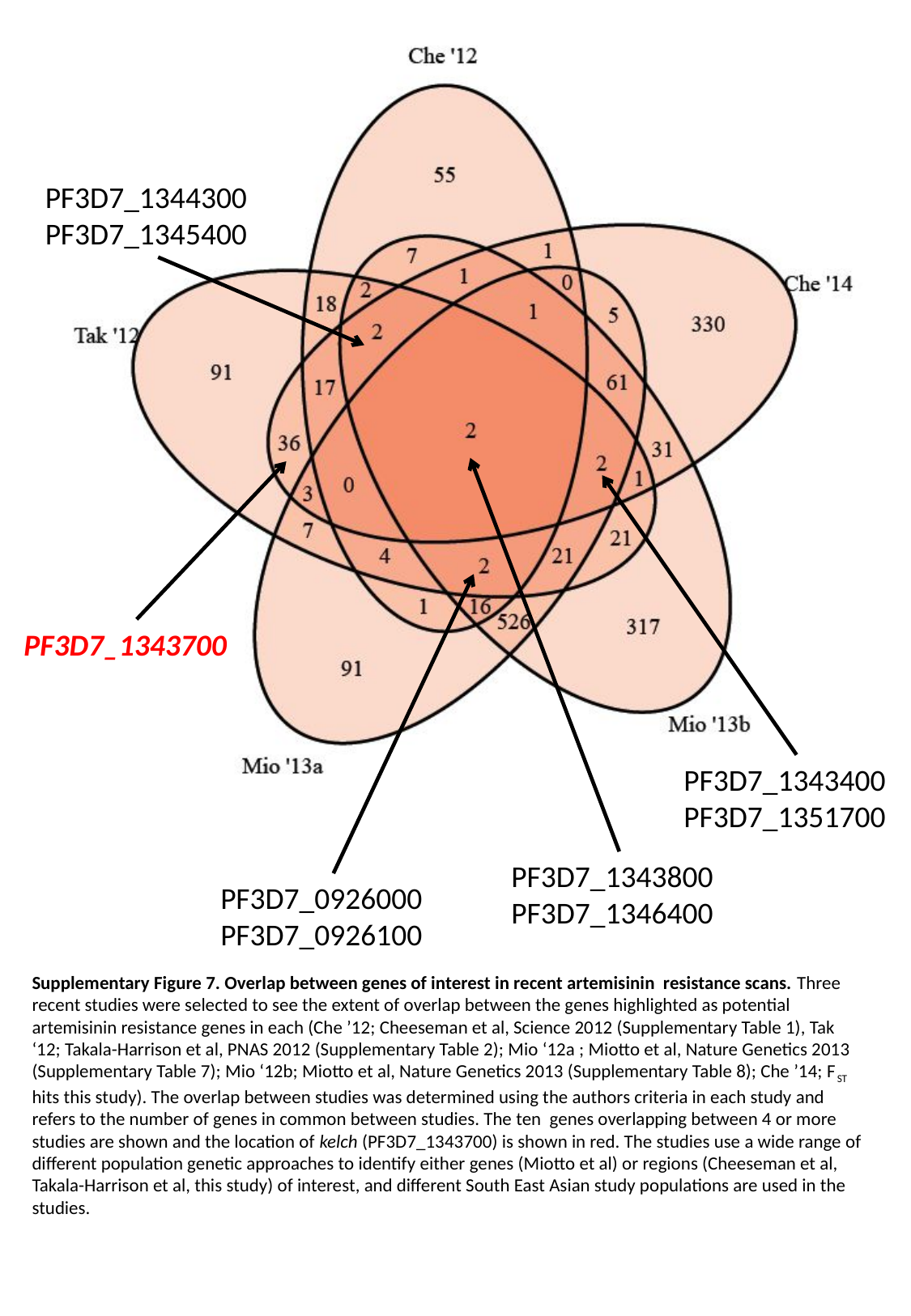

PF3D7_1344300 PF3D7_1345400
PF3D7_1343700
PF3D7_1343400 PF3D7_1351700
PF3D7_1343800
PF3D7_1346400
PF3D7_0926000 PF3D7_0926100
Supplementary Figure 7. Overlap between genes of interest in recent artemisinin resistance scans. Three recent studies were selected to see the extent of overlap between the genes highlighted as potential artemisinin resistance genes in each (Che ’12; Cheeseman et al, Science 2012 (Supplementary Table 1), Tak ‘12; Takala-Harrison et al, PNAS 2012 (Supplementary Table 2); Mio ‘12a ; Miotto et al, Nature Genetics 2013 (Supplementary Table 7); Mio ‘12b; Miotto et al, Nature Genetics 2013 (Supplementary Table 8); Che ’14; FST hits this study). The overlap between studies was determined using the authors criteria in each study and refers to the number of genes in common between studies. The ten genes overlapping between 4 or more studies are shown and the location of kelch (PF3D7_1343700) is shown in red. The studies use a wide range of different population genetic approaches to identify either genes (Miotto et al) or regions (Cheeseman et al, Takala-Harrison et al, this study) of interest, and different South East Asian study populations are used in the studies.
